# Editorial: Three-dimensional/3D stem cell culture systems

**DOI:** 10.3389/fcell.2023.1326727

**Published:** 2023-11-21

**Authors:** Katiucia Batista Silva Paiva, José Mauro Granjeiro, Leandra Santos Baptista, Alexandra P. Marques, Ana Rosa Ribeiro, Elizaveta Koudan

**Affiliations:** ^1^ Department of Anatomy, Laboratory of Extracellular Matrix Biology and Cellular Interaction, Institute of Biomedical Sciences, University of São Paulo, São Paulo, Brazil; ^2^ School of Dentistry, Fluminense Federal University, Niteroi, Brazil; ^3^ National Institute of Metrology, Quality and Technology (InMetro), Duque de Caxias, Brazil; ^4^ Nucleus of Multidisciplinary Research in Biology (Numpex-Bio), Federal University of Rio de Janeiro, Duque de Caxias, Brazil; ^5^ 3B’s Research Group—Biomaterials, Biodegradables and Biomimetics, Headquarters of the European Institute of Excellence on Tissue Engineering and Regenerative Medicine, University of Minho, Guimarães, Portugal; ^6^ ICVS/3B’s–PT Government Associate Laboratory, Braga, Portugal; ^7^ Nanosafety Group, International Iberian Nanotechnology Laboratory, Braga, Portugal; ^8^ National University of Science and Technology MISIS, Moscow, Russia

**Keywords:** stem cells, spheroids, organoids, microfluidics, organ-on-a-chip, 3D models, 3D bioprinting, bone

## Introduction

In the 20th century, the advent of cell culture techniques revolutionised the understanding of cellular mechanisms in physiological or pathological conditions. However, traditional bi-dimensional (2D) culture in a petri dish lacks important cell-cell and cell-extracellular matrix (ECM) interactions, culminating in an artificial microenvironment. In this way, cells embedded in three-dimensional (3D) matrices bring more cell interactions and improve cell response by using many materials to mimic the ECM, mainly collagens. Since the isolation and characterisation of the embryonic and adult stem cells and cell reprogramming, the modern 3D cell culture strategies try to recreate the tissue-specific organisation by self-assembling cells in a multicellular aggregates scaffold-based or scaffold-free way. In the last decade, the transition from traditional 2D to 3D cell culture increased substantially, and we can see massive progress in obtaining spheroids and very complex organoids and assembloids (Bissell, 2017; Jedrzejczak-Silicka, 2017). The European Association for Cancer Research (EARC), which coined the term “Goodbye Flat Biology,” initiated a series of conferences in 2014 to discuss the evolution of 3D microenvironment in the cancer context. Associated with the evolution of bioprinting techniques, we can now print structured tissue-like grafts for Tissue Engineering applications or develop new *in vitro* platforms for drug development or Precision Medicine. Also, dynamic cell culture (perfusion) methods from macro to micro scales boost the biophysical and biomechanical properties of the 3D constructions. This Research Topic presents six papers (reviews and original research) that will permeate all these subjects.

Over the last 70 years, different cell culture methods for skin reconstruction have been developed, such as 3D skin equivalents by cells-ladden in biomimetic matrices and, more recently, 3D bioprinting (Souci and Denesvre, 2021). Such models will be in demand not only for drug testing but also for testing cosmetics and household chemicals, modeling skin diseases, studies of absorption of substances through the skin and others. Here, Santos et al. compared the commitment potential of umbilical cord stem cells (a type of mesenchymal stem cell) into keratinocytes in a 3D *in vitro* skin model composed of dermal equivalent (collagen type I + dermal fibroblasts) or commercial porcine skin decellularised matrix, both in an air-liquid interface assay. The dermal equivalent induced more expression of epidermal markers and cell differentiation, resulting in skin organotypic cultures. This platform is a promise for skin biology studies and *in vitro* toxicological assays.

According to Atala’s classification of anatomical organs, skin is the simplest flat organ. This explains the many *in vitro* skin models and skin equivalents. Full-fledged models of more complex tubular and solid organs like the intestine, kidney or lung, are not yet available. The generation of miniaturised organs and tissues *in vitro* was possible after the crucial knowledge of pluripotent stem cells (embryonic and reprogrammed) and approaches for differentiation towards mature cell lineages. It gained force from the seminal study on gut organoid done by Sato et al. (Sato et al., 2009). Then, organoids will be applied to developmental biology, drug development, precision medicine, toxicology and disease modelling studies in various organs/tissues. Here, Xu et al. update the main advances and challenges in organoid models based on iPSCs (inducible pluripotent stem cells), such as the brain, liver, kidney, heart and lung (mainly to understand the mechanism of the COVID-19).

Cerebral and brain organoids, or “mini-brains,” were one of the first organoids made (human pluripotent stem cells) (Lancaster et al., 2013). Here, Horánszky et al. showed the effects of bisphenol A (BPA), a well-known endocrine-disrupting compound, in a new long-term human iPSC spheroid-induced neural stem cell differentiation model. BPA treatment (repeated-dose exposure with sub-cytotoxicity) reduced spheroid size and decreased cell proliferation but did not affect cells after 21 days in culture. Moreover, proteomic analysis confirmed these findings. It is a new method for investigating neurotoxicity in the earliest stages of central nervous system development.

The kidney is one of the most complex organs in terms of different cell types and multiple nephron segments. The first kidney organoid or tubuloid models have been established only from human embryonic stem cells (Takasato et al., 2014), iPSC (Taguchi et al., 2014), or adult/progenitor stem cells (Schutgens et al., 2019). Here, Hanhof et al. showed, for the first time, a duct-enriched 3D tubuloid from a mouse kidney primary cell culture, representing the collecting tube (CD), the most distal part of the nephron. This model forms a single layer of polarised epithelium in 3D. The upregulation of gene and protein expression of specific collecting duct markers, such as water channel AQP2 and the sodium channel ENaC, was confirmed. Also, the response to the pharmacological agents in calcium uptake has successfully assessed the physiological function. This model is a powerful tool for studying CD physiology and modelling many renal pathologies.

Bone is a complex organ which is composed of many different types of cells, including bone-forming cells (osteoblasts), bone-homeostasis cells (osteocytes), and bone-resorbing cells (osteoclasts). These cells reside on the surface or inside distinct bone architectures (trabeculae or cortical) and are exposed to internal and external stimuli. Also, bone is formed by organic (non-mineralised ECM—osteoid) and inorganic fractions (mineralised ECM with hydroxyapatite crystals) given different physiological microenvironments. In this way, the reconstruction of bone *in vitro* is very challenging, in which 2D cell culture brings poor results, and the primary way to study bone biology is *in vivo* models. Here, Lipreri et al. show how different 3D *in vitro* fluidic perfused platforms can mimic bone microenvironment at the macro/mili/microscale. These approaches will drive bone biology studies in the coming years.

Many advances have been achieved using 3D cell culture models, including organoids; however, they lack the physiological system in static cell culture mode. In this way, microfluidic devices can connect different organs/tissues on a microscale, bringing the microphysiology, named organ-on-a-chip. The first was developed in 2010 by recapitulating the lung alveolus (Huh et al., 2010). Here, Baptista et al. focused on converging organoid, 3D bioprinting and microfluidic technologies (named 3D organ-on-a-chip) to boost drug development and pharmacological screening.
